# Human synthetic lethal inference as potential anti-cancer target gene detection

**DOI:** 10.1186/1752-0509-3-116

**Published:** 2009-12-16

**Authors:** Nuria Conde-Pueyo, Andreea Munteanu, Ricard V Solé, Carlos Rodríguez-Caso

**Affiliations:** 1 ICREA-Complex Systems Lab, Universitat Pompeu Fabra. Parc de Recerca Biomedica de Barcelona, Dr Aiguader 88, E-08003 Barcelona, Spain; 2 Santa Fe Institute, 1399 Hyde Park Rd., Santa Fe, NM 87501, USA

## Abstract

**Background:**

Two genes are called synthetic lethal (SL) if mutation of either alone is not lethal, but mutation of both leads to death or a significant decrease in organism's fitness. The detection of SL gene pairs constitutes a promising alternative for anti-cancer therapy. As cancer cells exhibit a large number of mutations, the identification of these mutated genes' SL partners may provide specific anti-cancer drug candidates, with minor perturbations to the healthy cells. Since existent SL data is mainly restricted to yeast screenings, the road towards human SL candidates is limited to inference methods.

**Results:**

In the present work, we use phylogenetic analysis and database manipulation (BioGRID for interactions, Ensembl and NCBI for homology, Gene Ontology for GO attributes) in order to reconstruct the phylogenetically-inferred SL gene network for human. In addition, available data on cancer mutated genes (COSMIC and Cancer Gene Census databases) as well as on existent approved drugs (DrugBank database) supports our selection of cancer-therapy candidates.

**Conclusions:**

Our work provides a complementary alternative to the current methods for drug discovering and gene target identification in anti-cancer research. Novel SL screening analysis and the use of highly curated databases would contribute to improve the results of this methodology.

## Background

*High-throughput analyses *have provided a tremendous boost to massive drug screening [1]. However, these improved techniques are still blind to biological or structural knowledge. In this sense, *chemogenomics *provides a complementary strategy for a rational screening that includes structural information of chemical compounds for gene targets [2,3]. Computational approaches in this so-called virtual screening allow the matching of compounds to their specific gene-product targets, completing the experimental screening [4]. However, the computational approach is still limited by the huge combinatorics represented by the chemical space of possibilities associated to the compounds and their possible targets. As a consequence, all these experimental and computational approaches require the use of the cumulative biological knowledge. For this purpose, database integration into an *ontological *organization of the current biological knowledge has been suggested as a way to reduce the combinatorics either in virtual or experimental screenings [5]. The work presented here belongs to this last framework, intended as a tool for identifying potential targets for anti-cancer therapy. Cancer is a heterogeneous disease with numerous causes and typologies [6]. One of the essential traits of cancer progression is the underlying high mutational capacity of tumor cells [7-9], having as a consequence the rapid adaptive capacity of the disease. It has been suggested that these ingredients define cancer progression as a Darwinian micro-evolutionary process [10]. As a consequence, cancer cells which have lost essential genes by a mutation are eliminated from the tumor population. Therefore, it is expected that essential genes are conserved in cancer. Under this perspective, targeting essential genes in anti-cancer therapy could kill malignant cells, but might result to be lethal for healthy cells too. This is the case of the anti-proliferative drugs that also damage high turnover tissues, such as epithelium.

The problems reported from the failure of most single-target drug treatments [11] suggest that a new perspective is needed. In this context, a different conceptual framework related with synthetic lethality has been suggested for anti-cancer research [12-14]. Two genes are called synthetic lethal (SL) if mutation of either alone is not lethal, but mutation of both leads to death or a significant decrease in organism's fitness. According to screening methodology, two main types of mutations are considered: amorphic and hypormorphic mutations. The former causes a complete loss of gene function, while the latter refers to a mutation leading to a decreased activity in the respective gene function [15]. In genome-wide screenings of genetic interactions, hypomorphs are associated to essential genes such that the decrease of the gene expression does not result in inviable organisms [16].

**Figure 1:**
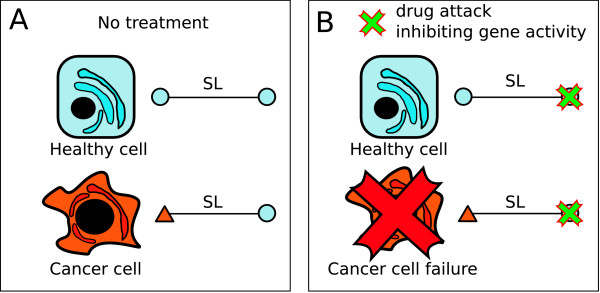
**The rationale of synthetic lethality applied to the design of novel anti-cancer therapies**. Two linked nodes (blue circles) represent a SL interaction. (A) In cancer disease, one of the SL partners would appear mutated (red triangle) contrasting to healthy cells where no mutation is accounted for. (B) The attack by drugs to the SL partner of the cancer-related mutated gene might cause a selective damage to tumor cells. In this case, the inactivation of *both *SL partners only happens in tumor cells.

The rationale of synthetic lethality offers new insights on selective anti-cancer therapy design by exploiting the existence of SL partners of mutated (cancer-related) genes [12,17,18]. Accordingly, given a mutated gene causing function deletion (amorphic mutation) or function decrease (hypomorphic mutation) in a cancer cell, an attack using specific drugs to block the activity of one of its SL partners would cause a lethal condition in such tumor cells. Meanwhile, only minor damage in healthy cells would be expected, constituting thus a selective anti-cancer therapy (see Figure 1). And thus, this approximation can help to overcome a dramatic limitation in drug design.

Another relevant aspect in drug screening is that one drug is tested only for a specific disease and related pathologies. Given a SL pair of genes as described above, one cancer mutated and the other non-mutated, conceptually it is possible that an already approved and even commercialized drug might block the activity of the non-mutated gene product. Therefore, SL-partner screening has a special interest for gene-target identification but also for drug repositioning, i.e, the discovering of novel uses for old drugs [19].

Unfortunately, large-scale screenings of SL gene pairs have been performed only in yeast [20-23] and, to a significantly smaller degree, in *C. elegans *[24-26] and in other model organisms. To overcome this limitation, we propose the use of the phylogenetic inference of SL genes from yeast to human for pharmacological purposes.

Synthetic lethal screens in yeast have been used to identify genes involved in cell polarity, secretion, DNA repair and cell cycle [27,28]. Due to the high conservation of genome integrity and cell-cycle related genes from yeast to higher organisms and their close relation with cancer disease [29], massive screenings of yeast SL interactions can provide a valuable information for SL inference applied to novel cancer therapy search. We emphasize though that the aim of this work is not to provide a general inference method of SL network from one organism to another. Instead, it adds to the rationale of drug design by supplying a candidate-list of human gene pairs, potentially SL, that could constitute the basis for future pharmacological testing. Additionally, network thinking has provided an excellent framework for the study of very large genetic systems. In particular, gene-disease [30,31], gene-target [32] and synthetic-lethal [23,33,34] network representations have contributed to the understanding of these systems as a whole. Following this approach, we integrate the information in a network framework from several databases in order to provide supporting evidence for candidates' reliability.

## Methods

**Figure 2:**
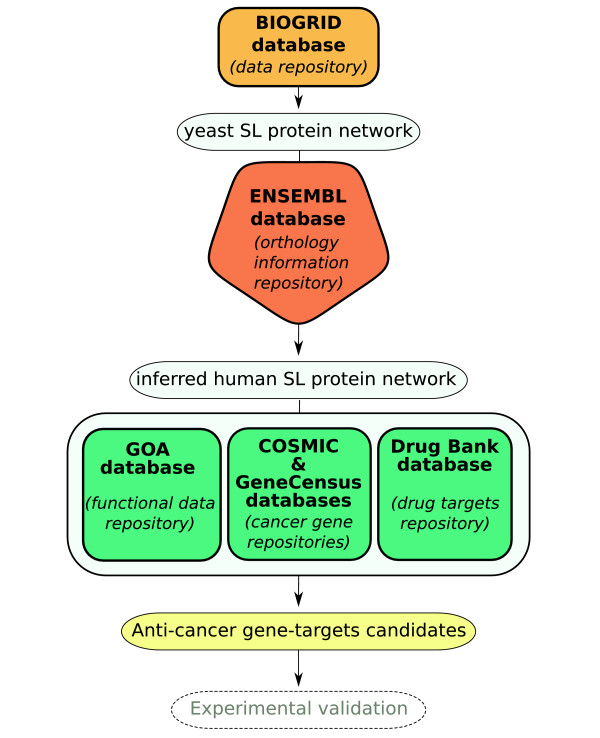
**Schematic representation of our methodology**. Potential anti-cancer gene-targets are proposed for future experimental validation.

Our identification of potential anti-cancer gene targets proceeds through the integration of the biological information originating from different databases. Figure 2 illustrates the methodology for the data-collection process and for the selection of potential anti-cancer gene targets. The yeast SL network was constructed from the yeast SL interaction list available from BioGRID database [35]. In this network, nodes represent genes and the link between them indicates a SL interaction, i.e. when both are simultaneously mutated, a lethal condition is satisfied. The phylogenetic inference from yeast to human genes was obtained from the Ensembl database noteEnsembl: http://ftp.ensembl.org/pub/current_emf/ensembl_compara/homologies, March 3^*rd*^, 2008. The yeast-genes list belonging to SL network was contrasted with this database. The inferred human SL network (in short iHSLN) is then obtained by introducing the yeast SL interactions on their human phylogenetically-conserved counterparts, that is their orthologs (see also [36]). Subsequently, as we detail below, the resulting network was filtered by different biological databases for public use: 1) COSMIC and Cancer Gene Census, 2) Gene ontology and Gene ontology annotation (GO and GOA) and 3) DrugBank database.

### Obtaining the yeast synthetic lethal network

The collected data on yeast genetic screens including synthetic lethality is available at BioGRID database (version 2.0.38) [35]. From this data, we retain only those genes having a systematic name as it appears in http://www.yeastgenome.org. The resultant compilation is derived from 1233 articles, with half of the total of 12707 SL relations being contained in five main articles [16,27,28,37,38]. As commented above, the data consists of knock-out and hypomorph experiments, with the latter corresponding to essential genes [16]. The list of essential genes has been downloaded from the Saccharomyces Genome Deletion Project http://www-sequence.stanford.edu/group/yeast_deletion_project/deletions3.html.

### Obtaining the iHSLN

**Figure 3:**
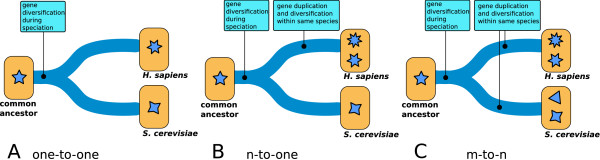
**The orthology relation in the process of yeast-to-human inference method**. The *one-to-one *case of one yeast gene having one human homolog (A). The *n-to-one *case of several human genes having the same yeast homolog (B). The *one-to-m *case can be defined analogously. The *n-to-m *is a very rare case where more than one human gene are homologs of more than one yeast gene (C).

**Figure 4:**
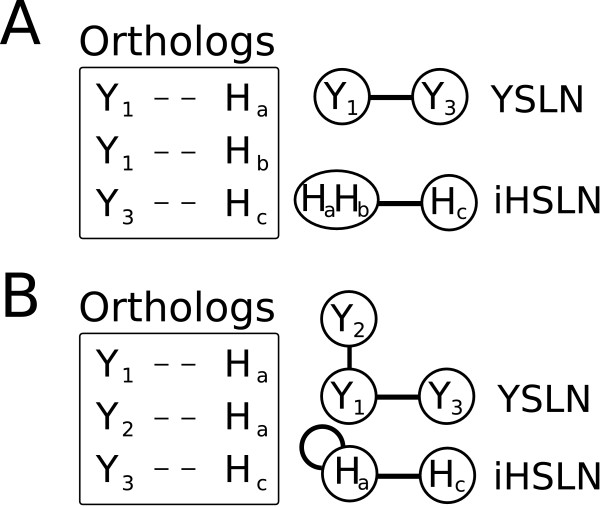
**Gene-node convention for the iHSLN construction**. Every node in yeast SL network (YSLN) has associated gene. (A) For *n-to-one*, the corresponding node in the iHSLN contains the human paralogs of the yeast homologous counterpart. (B) In the case that two SL yeast partners are phylogenetically related to a single human gene, an autolink appears in the human SL network. This is and artifact of the method. However, we have kept this information to provide a more detailed picture in case such a node is a suitable candidate.

**Figure 5:**
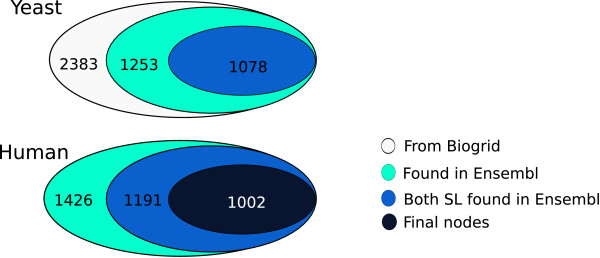
**The statistics of the genes vs nodes the from gene-inference process**.

Homologs between *H. Sapiens *and *S. cerevisiae *were obtained by similarity measures from the whole-genome multiple alignments by the Ensembl comparative genomics team. Human genes in this study were named according to HUGO gene nomenclature committee (HGNC http://www.genenames.org/). Each entry of the homology data file contains gene's evolutionary history corresponding to a gene tree that diverged from a common ancestor (see Figures 3 and 4). In our study, gene conservation in yeast and human can be summarized in three types of relations. The simplest case corresponds to *one-to-one *relation between yeast and human genes (orthologous relation). However, duplication events during evolution led to two alternative cases (see Figure 3B), where one yeast gene has more than one human homolog (*one-to-m *relation), and vice versa (*n-to-one *relation) [36]. The case of *n-to-m *was rarely encountered (Figure 3C). A yeast-human inference relation is straightforward for the *one-to-one *cases, and the human gene inherits the SL neighbors of its yeast homolog. On the contrary, the paralog cases require the introduction of approximate relations. More precisely, we manually curated all the cases where the inference is not *one-to-one*. For the *one-to-m *cases, we collapsed the multiple human genes into a single node that inherits the SL links of the yeast ortholog (Figure 4A). This situation does not affect the pattern of interactions derived from yeast SL network. In addition, by checking the biological function of these *m *human genes, we classify them into subsets of similar functions, and collapse these subsets into separate nodes. Thus a yeast *gene *having *m *orthologs might correspond to more than one *node *in the human SL network. Again, we emphasize the distinction between the single-gene nodes in the yeast SL network and the potentially multiple-gene nodes in the iHSLN. The statistics of the genes vs nodes the from gene-inference process is illustrated in Figure 5.

The *n-to-one *cases merge the SL information from the multiple yeast nodes, and therefore a single human gene inherits the SL interactions from more than one yeast gene. The extreme case is the third one, *n-to-m*.

### Gene function statistical analysis of the iHSLN

Significance of the biological-function representation in iHSLN was evaluated by means of a hypergeometric test using Benjamini & Hochberg false discovery rate with a P-value < 0.05 as a minimal cut-off. All the genes belonging to iHSLN, independent on whether they belong to multi-gene nodes, were considered in the analysis. Only eight genes were not associated to any GOA term. Statistics were performed for biological-function GOA terms using BINGO 2.3 Cytoscape plugging [39]. A detailed report including the list of genes related to specific GOA terms is available at Additional File 1.

### Filtering the iHSLN

Biological filtering through the use of databases led to the functional classification of the nodes forming the iHSLN into: (1) cancer-related genes, (2) genes related to the DNA repair mechanism and cell cycle (two relevant functions altered in most cancers) and (3) drug-target genes.

In order to identify cancer-related candidates we use the COSMIC database(COSMIC: http://www.sanger.ac.uk/genetics/CGP/cosmic: 37^*th *^version) that stores the current knowledge on somatic mutations and related details on human cancers. The information was completed by the use of the genes from the Cancer Gene Census http://www.sanger.ac.uk/genetics/CGP/Census that is a project to catalogue those genes for which mutations have been causally implicated in cancer.

Gene Ontology (GO http://www.geneontology.org, April 6^*th*^, 2008) database provides the biological description of gene products for a number of predefined functions. To relate one gene with its GO annotation, we used the gene ontology annotation (GO numbers) from GOA http://www.ebi.ac.uk/GOA/ 8^*th*^,2008). We also used the available relationships between GO terms [40] to create different *filters *for different biological processes, in particular for Cell cycle and DNA damage.

In addition to these databases, we have also used the information extracted from the DrugBank (http://www.drugbank.ca, release 2.0). It provides the available studies from the US Food and Drug Administration that relate tested drugs and their gene targets [32,41,42]. In this way, we inquire whether clinical studies of drugs acting on our proposed candidates have been previously described in literature. All the representations of the resultant networks were performed with Cytoscape [43]. Networks are provided as additional files.

## Results

### The iHSLN

Yeast SL network is a graph of 2383 nodes and 12707 links. This network is an extension of the SL network constructed by [28], who remarked that SL interactions yield a giant component with a non-random topology of small-world characteristics.

According to Ensembl database, phylogenetic inference revealed that 52% of yeast SL nodes (1253 of 2383 SL yeast genes) has at least one putative human ortholog (see Figure 5).

**Figure 6:**
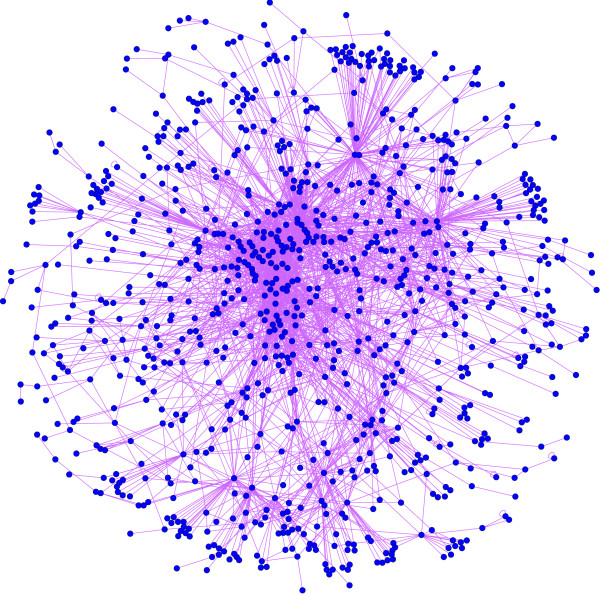
**The 933-giant component extracted from the 1002-nodes network of inferred SL human genes **(Additional files 4 and 5). The human orthologs from yeast are related through a yeast SL-relation.

Previous analysis of global genomic homology between species estimated that the coverage between *S. cerevisiae *and *H. sapiens *is about 20% [44]. The higher percentage observed from the set of the yeast SL genes compared with the global coverage may suggest a possible conservation for SL proteins. However, it is also worth noting that a criterion for gene selection in SL screenings is precisely a high conservation along evolution, and this could be the cause of the high proportion of inferred human genes in the SL set. From the above-mentioned 1253 genes, only 1078 genes have a SL partner, that is those genes among the 1253 that have SL partners without any human ortholog are eliminated (Additional file 2). The final human SL network presented a set of 1002 nodes and 2847 links (Figure 6 and Additional files 4 and 5), where almost all the nodes formed a giant component (933 nodes) with a non-random topology. Considering yeast-to-human inference relations, the nodes in the human network resulted from 766 *one-to-one *homology cases, 64 human nodes are composed of 138 genes due to the *n-to-one *cases, and 170 nodes are formed of 386 genes due to *one-to-m *relations (more precisely, 148 nodes include gene of similar biological function, while 21 nodes contain genes with heterogeneous functionality). Only 2 nodes came from 2 *n-to-m *situations. We mention that 1.5% (43 nodes) of the links in the human network were autolinks (see Figure 4B for explanation). This is a result of the *n-to-one *cases, as among these *n *yeast genes some may be SL partners. This is an artifact of the human SL network. These autolinks in the iHSLN are not eliminated from our network, as the discussion concerning these nodes as candidates needs to be addressed with caution. We stress once more that in our subsequent analysis we refer to nodes rather than to simple genes, as a node may contain several genes.

Statistical analysis of biological-function representation in iHSLN indicated a marked significant overabundance (P-value ≪ 0.05) of genes related to genome integrity and cell cycle among others (see Table 1). This fact is explained by the enrichment of genes related to DNA integrity functions due to the selection criterion applied to yeast SL screenings, enrichment that holds in iHSLN too by the high conservation of these genes. As previously pointed out, such conserved functions in iHSLN are closely related to cancer disease providing a valuable set of potential SL candidates for humans.

**Table 1:** Statistical analysis of iHSLN biological functions.

*GO term - Biological function*	*P-value*	*% of genes in iHSLN*	*% of genes in GOA*
DNA replication	5.20 E-28	6.8	40.9
DNA repair	4.93 E-24	7.4	27.7
Response to DNA damage stimulus	4.00 E-23	8.1	24.9
RNA processing	4.56 E-15	8.7	17.9
Cell cycle	2.50 E-13	9.8	15.7
Proteasomal protein catabolic process	1.10 E-8	24.0	30
Golgi vesicle transport	1.70 E-8	25.0	28.4

The columns include: Column 1 - the biological function from GOA; column 2 - the P-value associated to the results; column 3 - the percentage of genes with a given GO term encountered in iHSLN (998 genes); column 4 - the percentage of genes with a given GO term encountered in GOA (14486 genes) (see Additional file 1).

### Obtaining potential cancer-related SL targets

#### Cancer-related database approach

**Figure 7:**
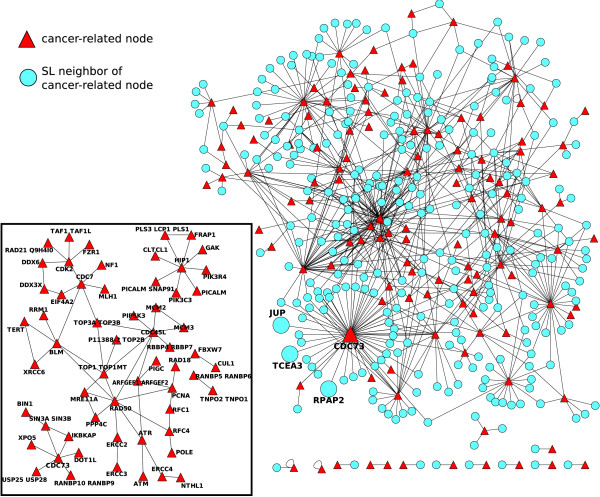
**Cancer-related genes and their first neighbors from the SL human network **(Additional file 6). The inset illustrates the sub-network of cancer-related nodes alone (59 nodes). Nodes without SL partners (65 of 124) were eliminated from this picture. In the large network, both cancer-related (triangles) and their neighbors (circles) in the SL human network. Only the links connecting a cancer-related gene and its neighbor (411 nodes and 694 links) are illustrated.

In order to evaluate potential candidates, we first identify those human genes in our inference list that are known to be involved in cancer mutations, as detailed in Material and methods. Given this information, the SL partners of these genes are then potential candidates for anti-cancer therapy. In the inset of Figure 7 we represent the 124-nodes sub-network containing those nodes in the human SL network that have been found in the COSMIC and Cancer Gene Census databases. In this network, we observe the SL interactions between cancer-related genes. However, our objective is to obtain possible candidates, that is the SL partners of these 124 that are not described as cancer-related genes. Therefore, we extract from the iHSLN also the first neighbors of these 124 nodes. We illustrate this network in Figure 7 where triangles depict cancer-related nodes and circles indicate their neighbors that are not known to be cancer-related in the used databases. This figure represents a map of the potential SL candidates to be targeted when a given cancer-related mutation is predominant in a tumor. To evaluate the significance of cancer-cancer correlation in iHSLN, we performed a randomization with 5000 runs of the cancer-node attributes maintaining the topology of the network from Figure 7. The statistical test using t-student revealed that cancer-cancer links are significantly overabundant in the iHSLN.(Additional file 6)

A word of caution is required before interpreting Figure 7. Cancer-related information is a compendium obtained from many samples, cancers and types of mutations. Given a SL pair conserved in human, if amorph (or even hypomorph) mutations occur in both genes within a tumor cell then a lethal condition should be expected according to our rationale. Following this reasoning, such a combination of mutants should not be observed within a cell. If such an event results in Figure 7, we would state that this combination is not conserved in humans according to SL definition. Therefore, we interpret cancer-cancer SL interactions to occur in different samples.

Notice that the cancer-related databases include both those genes that are also overexpressed in tumor cells. Even though the expression level information is not always available, this information is determinant for a correct selection of SL candidates. The most likely candidates presented in this work are chosen according to an underexpression scenario. In relation with this issue, among the many types of mutations encountered in the databases we wish to single out a few examples of mutations types. Some cancer-related genes in Figure 7 correspond to cases of nonsense mutations, in other words point mutations causing a premature stop codon. Therefore, we expect that such mutations critically damage the function of corresponding proteins. These genes are ATM, NF1, FBXW7, MSH2, BUB1, ERCC2, BLM and MSH6. The first four genes are also documented in the Cancer Gene Census to suffer deletions in different types of cancers.

Providing a proof of the potential predictability of our iHSLN is not straightforward due to the sparseness of data and the lack of a systematic identification of SL in humans. However, a number of examples can be extracted from literature, supporting the utility of our approach. One of them is the above cited BLM gene. It is a highly connected gene with 24 SL partners in the iHSLN that codifies a helicase homolog to SGS1 yeast gene. Its deficiency causes the Bloom syndrome, an autosomal recessive disorder with high disposition to tumorigenesis process. In a work performed in Drosophyla, BLM-MUS81 double mutation is an experimental verification of a SL interaction observed in our iHSLN [45]. This fact has a special interest since BLM helicase facilitates MUS81 endonuclease activity in human cells [46]. More recently, a SL interaction between the above cited NF1 gene and RAD54B, an homologous of the yeast RAD54, was experimentally determined in cancer cells [47]. Although yeast screenings identified this double mutant as a SL pair, due to database incompleteness, the phylogenetic relation of RAD54 was lost during inference process. As we discuss later, the quality of data is a considerable pitfall for a good performance of this methodology.

Another indirect evidence of a potential verification comes from the lethal effect of ATM gene deletion in defective Fanconi anemia (FA) pathway cells. FA pathway inactivation is strongly associated to tumorogenesis process. Interestingly, an activator of this pathway is ATR, a SL partner of ATM in the iHSLN [48].

One of the hubs observed in Figure 7 corresponds with CDC73. It has been observed that mutations in this gene (also known as HRPT2) are associated with malignancy in sporadic parathyroid tumors and hereditary hyperparathyroidism-jaw tumor syndrome [49]. Parafibromin is a tumor suppressor protein encoded by HRPT2 that binds to RNA polymerase II as part of a PAF1 transcriptional regulatory complex. Expression inhibition by HRPT2 RNA interference stimulates cell proliferation and increases the levels of the c-myc proto-oncogene product [50]. According to Figure 7, the CDC73 hub establishes a large number of potential SL candidates. We must stress that, due to the long phylogenetic distance between human and yeast, some of them are likely false positives. Nevertheless, biological arguments in favor of their relevance can be sought in order to strengthen their candidacy. This is the case of the SL link between CDC73 and genes related with the RNA polymerase machinery such as RPAP2 [51], and the transcription elongation factor TCEA3 [52] that capture the common function in RNA processing of these partners. In addition, CDC73 has as SL partner in Figure 7 the JUP (junction plakoglobin or *γ*-catenin) protein, a component of the catenin complex. JUP is involved in cell adhesion [53], an apparently very distant function regarding RNA processing. However, *γ*-catenin strongly activates the proto-oncogene c-myc [54]. According to this evidence, it seems reasonable to consider that a biological pathway relates JUP and CDC73 (depicted as SL partners in our study) with c-myc, but with opposite effects. In this case, our methodology reveals a potential anti-cancer strategy: inhibiting *γ*-catenin expression would compensate the activation of c-myc by the HTRP2 cancer mutation.

#### GOA filtering approach

In a second approach, and in agreement the to statistical significance of the functional analysis (notably, all GOA terms presented in Table 1 produced a very significant P-value), we added a new layer of information by using the cancer-related functions extracted from GOA. In so doing, we searched for candidates that might not be included in cancer-related databases, but their functions are essential for cancer progression. These functions have an special interest due to, it is well established that mutations in genes involved in DNA damage repair or in cell cycle checkpoints are associated with tumor progression [55]. In this sense, synthetic lethal relationships between DNA-replication genes (such as certain DNA polymerases) and DNA-repair genes (such as mismatch-repair genes) are well documented in model organisms [29,56]. Moreover, it seems likely that the efficiency of many anti-cancer drugs that interfere with DNA synthesis is in some cases due to the presence of tumor-associated mutations affecting DNA repair or the response to DNA damage [18].

**Figure 8:**
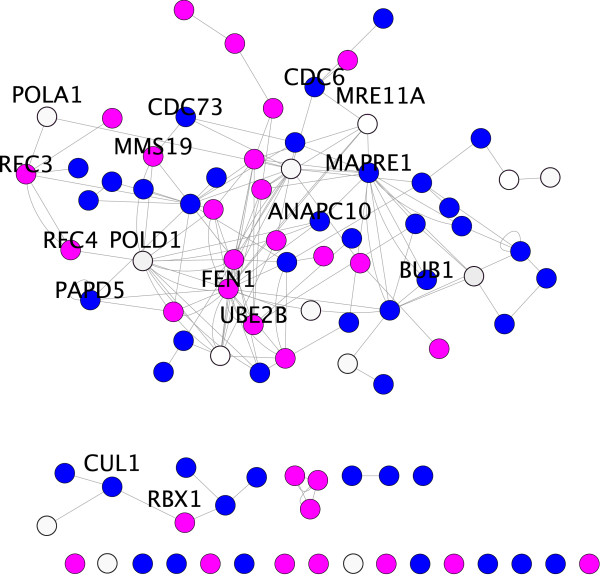
**SL-inferred human orthologs organized by GO numbers **(Additional file 7). Genes' color denotes their biological function (GO number): in blue, cell-cycle related genes, in red, DNA damage related genes and in white, genes related to both processes.

The connected sub-network from Figure 8 (Additional file 7) was obtained by extracting the genes annotated for these functions. Interestingly, the analysis shows that for six SL pairs one gene is directly cancer-related, whereas the other is not known to be so (Table 2). These latter genes constitute preliminary and putative candidates for future experimental validation. It is also worth noting that, as shown in Table 2, we also consider as candidates some SL partners with no known relation to cancer. They are SL partners consistent with a *within pathway *approach [23] (they act, or are part of the same complex in a pathway). Moreover, we notice that these cancer-unrelated SL pairs (labeled with asterisk in Table 2) consist of essential genes and thus come from hypomorph experiments. Their essential nature strengthens the likelihood of their belonging to the within-pathway model rather than to the *between-pathway *one (they function in parallel pathways).

**Table 2:** Yeast SL gene-pairs.

*Yeast*	*Human*	*Biological process*
YMR224C	**MRE11A**	Single-strand and double-strand-3'-5' exonuclease activity for DNA repair and recombination.
YER016W	MAPRE1	Microtubule assembling

YLR418C	**CDC73**	PAF1 complex. Interacts with RNA pol II. Causes hyperparathyroidism-jaw tu- mor syndrome
YIL128W	MMS19	Interacts with helicase subunits of NER-transcription factor

YNL299W	**PAPD5**	Sister chromatid cohesion. Resistance to Campothecin anti-tumor agent
YJL115W	ASF1	Histone chaperone

YJL194W	**CDC6**	Initiation of DNA replication. Oncogenic activity through repression of INK4/ARF locus
YGL087C	UBE2V2	Ubiquitin-conjugating enzymes without catalytic activity

YJL013C	**BUB1**	Involved in cell cycle checkpoint enforcement. Colorectal cancer
YGL058W	UBE2B	Central roll in postreplicative DNA repair in eukaryotic cells

YKL113C	**FEN1**	Removes 5' overhanging flaps in DNA repair and Okazaki fragments. Fast tumor progress
YGL240W	ANAPC10	Component of anaphase promoting complex (APC), progression through mitosis and G1 phase

*YDL132W	CUL1	Core component of multiple cullin-RING-based SCF which mediate the ubiquitination of proteins involved in cell cycle progression, signal transduction and transcription.
YOL133W	RBX1	Component of the SCF.

*YBR087W	RFC3	form a complex required by DNA polymerase delta and epsilon.
YNL102W	POLA1	DNA polymerase subunit.

*YJR068W	RFC4	form a complex recruited by DNA polymerase
YDL102W	POLD1	DNA polymerase subunit

Human homologs of the SL gene-pairs and the biological process that they fulfill. A preliminary candidates list, where a human gene is cancer-related (in bold) and its SL partner is not known to be directly related with cancer. Asterisk symbol indicates SL partners that belong to the same cellular pathway (*within-pathway *model: [23]) and are essential.

An illustrative case by means of the GOA filtering is again CDC73, also observed in the previous section. In this case, GOA filtering offers evidence of its relation with the DNA damage related gene MMS19 (see Table 2) and helicase component. Interestingly, helicases have been proposed as targets for anti-cancer therapy [57] as they are closely related with the required genetic instability for tumor progression.

#### Drug association to SL human target genes

DrugBank database information was used here to establish possible relations between existing drugs and inferred SL human genes. We have found that 130 nodes of our iHSLN contain genes associated to one or several drugs in the DrugBank. More specifically, 17% of them are anti-cancer drugs. See Additional file 3 for pattern of interactions for this set of nodes. As we shall see in the next section, the combination of cancer-related and drug-target information into the iHSLN produces a set of genes that are known drug targets and have cancer-related SL partners, i.e. the suitable candidates according to our methodology illustrated in Figure 1.

#### Finding SL partners of cancer-related genes associated to drugs

**Figure 9:**
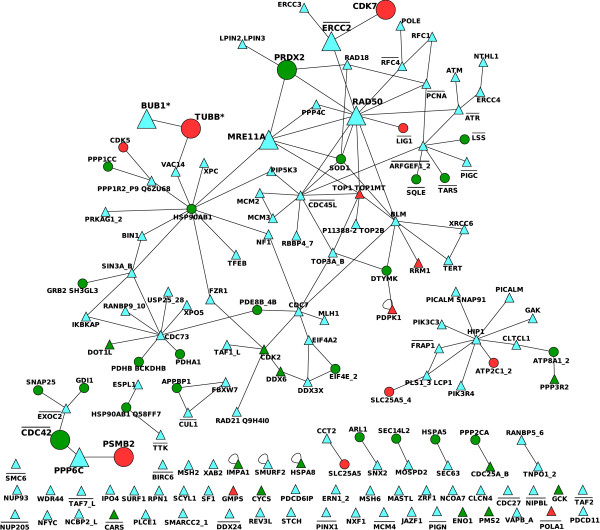
**Drug-cancer SL network representing the set of cancer-related nodes and their SL drug-associated partners **(Additional file 8). Triangles represent cancer-related nodes and circles, SL drug-associated partners. Red color indicates that this node has associated a gene which is target of anti-cancer drug. Green color indicates a non-cancer drug associated to the node. Blue color only appears in triangles representing that there is no drug in the DrugBank database associated to these cancer-related genes. Essential genes are overlined. Large nodes are examples of existent anti-cancer drug target with a cancer-related SL pair (PPP6C-PSMB2, BUB1*-TUBB*, ERCC2-DCK7), and also our suggested examples of novel use for existent cancer-unrelated drugs that target a SL partner of a cancer-related gene (RAD50-PRDX2, MRE11A-PRDX2 AND PPP6C-CDC42). The BUB1* node represents BUB1 and BUB1B genes, whereas TUBB* node includes TUBB2B, TUBB3, TUBB2A and TUBB6 tubulin family members.

As we previously argued, SL partners of cancer-related genes constitute a set of potential targets for anti-cancer treatment. The knowledge about drugs affecting these genes provides, on one hand, supporting evidences of our methodology. This is the case of cancer-unrelated genes for which there exists an anti-cancer drug, genes that are also SL partners of a cancer-related gene. On other hand, when the cancer-unrelated gene is associated to a cancer-unrelated drug, we have a potential new use of a drug that initially has not been conceived for anti-cancer treatment. The integration of this information is included in Figure 9 (Additional file 8). In this network, we eliminated the cancer-unrelated nodes that are not associated to any drug. The remaining set contains 155 nodes of which 124 are cancer-related nodes and 31 are drug-target nodes. A subset of 47 nodes are isolated nodes that match the number of cancer-related nodes having lost all the SL partners during filtering process, and it also indicates that no drug is associated to their SL neighborhood in the current version of DrugBank. Moreover, we notice that 28 nodes of 155 correspond with essential genes in yeast, according to Saccharomyces Genome Deletion Project http://www-sequence.stanford.edu/group/yeast_deletion_project/deletions3.html. Randomization and the subsequent statistical test using t-student revealed that cancer-to-drug-target links are significantly overabundant in the iHSLN.

Figure 9 also singles out (large circles and triangles) three SL pairs that are fundamental to existent anti-cancer treatments consisting in targeting SL partners of the cancer-related genes. In the first example, ERCC2 (also known as XPD) is a cancer-related gene [58]. Studies of its homologous gene in Drosophila show that an excess of XPD produces a titration of CAK complex and reduces CDK7 activity, leading to a cell-cycle arrest [59]. Moreover, CDK7 is a protein of the CAK complex required for cell-cycle progression [60]. It has already been suggested as a potential target for anti-cancer therapies. Pharmacological implications are discussed in [60].

In the second example, PPP6C is a phosphatase involved in cell-cycle progression in human cells through the control of cyclin D1 [61]. Its SL partner, PSMB2, is a proteasome subunit related with protein degradation processes. Bortezomib, a therapeutic proteasome inhibitor is a target of PSMB2 (according to DrugBank information). It is pharmacologically approved for treating relapsed multiple myeloma and mantle cell lymphoma. In addition, the use of Bortezomib in hepathocarcinoma cells reduces the transcriptional levels of cyclin D1, among other effects, leading to cell-cycle arrest. It is postulated that cyclin D1 can act as an oncogene [62,63]. In this case, we observe that PSMB2 and PPP6C affect the levels of this cell-cycle related gene.

The third example, BUB1* (here the * symbol implies that more than one phylogenetically-related proteins is contained in this node) is a prototype member of a family of genes, some of which encode proteins that bind to the kinetochore and all of which are required for a normal mitotic delay in response to spindle disruption. Mutations in this gene have been associated with aneuploidy and several forms of cancer [64]. Our results reveal that this gene is associated to TUBB* in the SL network. TUBB* represents a number of genes encoding tubulin proteins. Tubulins are targets of the anti-cancer drug Paclitaxel and the vinca alkaloids Vincristine and Vinblastine that also affect the mitotic-spindle assembly process [65].

Table 3 contains a representative list of the assignment of a potential anti-cancer use for existent cancer-unrelated drugs. As examples, two genes (and their drugs) are commented. The first one is PRDX2 (also known as peroxiredoxin-2), a gene encoding a member of the peroxiredoxin family of antioxidant enzymes, which reduce hydrogen peroxide and alkyl hydroperoxides [66]. It is interesting to mention that PRDX2 has been associated to cell proliferation and cell migration by regulation of the PDGF signalling [67]. N-carbamoyl alanine and 3-sulfinoalanine compounds were found to be inhibitors of PRDX2 activity and it has not been yet associated to any anti-cancer treatment. Two SL cancer-related partners of PRDX2, RAD50 and MRE11A are part of MRN protein-complex involved in DNA double-strand break repair, cell-cycle checkpoint activation, telomere maintenance and meiotic recombination [68].

**Table 3:** A selection of cancer-unrelated SL partners of cancer-related genes and their associated drugs.

*Drug-target name*	*Drug (Description)*
CDC25B	Beta-Mercaptoethanol (Glutathione S-Transferase inhibitor); Cysteinesulfonic Acid(Peroxiredoxin inhibitor); Double Oxidized Cysteine (Peptide Deformylase Pdf1 inhibitor)
CDC42	Aminophosphonic Acid-Guanylate Ester (G25K GTP-Binding Protein inhibitor)
CDK2	4-(2,4-Dimethyl-Thiazol-5-Yl)-Pyrimidin-2-Ylamine (Cell Division Protein Kinase 2 inhibitor)
DDX6	D-tartaric acid (D-tartaric acid)
EIF4E	7-Methyl-Gpppa (Eukaryotic Translation Initiation Factor 4E inhibitor); 7n- Methyl-8-Hydroguanosine-5'-Diphosphate (Vp39 inhibitor)
GDI1	Geran-8-Yl Geran(Rab GDP Disossociation Inhibitor Alpha inhibitor)
GRB2	4- [(10s,14s,18s)-18-(2-Amino-2-Oxoethyl)-14-(1-Naphthylmethyl)-8,17,20-Trioxo- 7,16,19-Triazaspiro[5.14]Icos-11-En-10-Yl]Benzylphosphonic Acid (Growth Factor Receptor-Bound Protein 2 inhibitor)
HSP90AB1	9-Butyl-8-(3,4,5-Trimethoxybenzyl)-9h-Purin-6-Amine (Heat Shock Protein Hsp 90-Beta inhibitor)
HSPA5	antihemophilic factor (Coagulation factor VIII precursor)
LSS	Dihydrofolic Acid (Dihydrofolate Reductase inhibitor)
PPP1CC	9,10-Deepithio-9,10-Didehydroacanthifolicin (Serine/Threonine Protein Phos phatase Pp1-Gam inhibitor)
PPP2CA	Vitamin E (Dietary supplement)
PPP3R2	Cyclosporine (Investigational Immunomodulatory Agents; Immunosuppressive Agents; Antifungal Agents; Dermatologic Agents; Enzyme Inhibitors; An tirheumatic Agents For treatment of transplant rejection, rheumatoid arthritis, severe psoriasis)
PRDX2	3-Sulfinoalanine (3-Hydroxy-3-Methylglutaryl-Coa Synthase inhibitor)
SEC14L2	Palmitic Acid (Enzyme Inhibitors)
SNAP25	Botulinum Toxin Type A (Anti-Wrinkle Agents; Antidystonic Agents; Neuromuscular Blocking Agents)
SOD1	S-Oxy Cysteine (Prolyl Oligopeptidase inhibitor)
SQLE	Naftifine (Anti-Inflammatory Agents, Non-Steroidal; Antifungal Agents)

These drugs are not oriented to anti-cancer treatment. In this list we excluded general metabolites described as dietary supplements in DrugBank. A short description of drug activities are provided in parenthesis.

The second example is the SL link between CDC42 and the previously described PPP6C. CDC42 is a gene participating in the rearrangement of actin cytoskeleton, membrane trafficking and cell-cycle progression, and it appears to be involved in cardiovascular diseases, diabetes and neuronal degenerative diseases [69]. This is a target gene of two other compounds (aminophosphonic acid-guanylate ester and guanosine 5'diphosphate) with no relation to cancer treatment in the DrugBank. However, it has been described that this gene is also involved in tumorigenesis and tumor progression, and the aberrant expression of CDC42 has been associated to colorectal tumors [69,70]. It is worth mentioning that it has been observed that CDC42 controls the cell growth of anaplastic large cell lymphoma through its activation. Pharmacologic inhibition of CDC42 activity by secramine results in a cell-cycle arrest and apoptosis of these cells [71,72]. We suggest thus that CDC42 inhibition by secramine constitutes a potential anti-cancer treatment, but unfortunately neither CDC42 nor secramine appeared in their respective databases used in this study. This example emphasizes once more that database information is a useful starting point for selecting new candidates. Interestingly, yeast ortholog of CDC42 is an essential gene and thus its mutant in the screening experiments is a hypomorph. Thus it suggests that partial inhibition of CDC42 could be enough to cause a lethal condition in those tumor cells where an amorphic mutation for PPP6C exists. In view of these comments, novel SL screening analysis and the use of highly curated databases would contribute to improve the results of this methodology.

## Discussion

By the present study we propose a methodology for providing liable candidates for future experimental validation as drug targets for anti-cancer therapy. The methodology is based on the existence of synthetic-lethal relation between pairs of genes: two genes are synthetically lethal if their simultaneous mutation leads to inviable organism, while their separate mutation has no substantial effect on the organism's fitness. As conceptualized by previous works [17,18], we used here the extensive experimental data on yeast in order to extend the knowledge to the human genome, and more precisely to anti-cancer therapy.

The rationale behind such approach is that, assuming that there are specific mutations for cancer cells, the identification and artificial mutation (drug action) of their SL partners would result in the death of cancerous cells alone. The mutations would affect also healthy cells, but would not drastically injure them. The combination of different biological databases provides potential filters for reducing the number of false positives. In this work a gene is considered cancer-related if it belongs to COSMIC or Cancer Gene Census databases. This implies certain limitations to our study since it depends on the accuracy and completeness of these databases. In this context, the better annotation of the used database, the more reliable the results. One example of that is the case of CDC42-PPP6C SL pair and the use of secramine drug resulting from a literature search but not from the current DrugBank version.

We have commented that cancer-cancer SL interactions could be interpreted as false positives as we do not expect them to occur in the same cell. We consider that the proof of this statement is an interesting working hypothesis to be tested in future research. Such a future research would aim at providing a quantitative estimation about the likelihood of observing a double mutation in tumor cells as it results from the data analysis. A supporting evidence of this hypothesis would be that SL cancer-related gene pairs are less likely to be observed in the double-mutation dataset. In spite of being related to the current work, we consider such a study to be outside the aim of the current work directed at introducing the potential of this methodology.

In addition, false positives can result also from the long evolutionary distance and different architectures between yeast and human genomes. At this point, the evolutionary conservation of SL pairs is controversial [29,73-75], even though a recent study inclines the balance towards a significant conservation of synthetic lethal interactions between eukaryotes [75]. In addition, the existence of conserved SL associated to particular functions [29,74,76] is promising evidence for the inference methodology presented here. Even though not all yeast SL pairs are expected to be conserved in distant organisms, those associated to essential functions have a higher conservation probability. The identification of only a few of such partners could constitute alone an invaluable information in the strategy of drug design.

As Lawrence Loeb stated with his mutator-phenotype scenario for cancer evolution [7,9,77], some genetic instability but not too much is required for cancer progression. An illustrative example, the BUB1-TUBB* SL pair, is closely related with the strategy of forcing instability in order to kill cancer cells. It is reasonable to assume that an attack to tubulins by drugs in tumors where BUB1 appears mutated may drift the tumor population towards extinction by exceeding the limits of mutation tolerance. In this particular case, we speculate that treatments with vinca alkaloids should be more efficient in those cancers where BUB1 is mutated. Analogously, our results reveal the suitability of an attack to MMS19 helicase component when CDC73 is mutated. As argued by [78], compensatory helicase-dependent DNA repair pathways may represent a suitable target for anti-cancer therapy strategies that are designed to introduce DNA damage to tumors with pre-existing defined DNA repair deficiencies. In this context, we provide among our candidates the FEN1-BLM pair and the already confirmed MUS81-BLM [45], both suggested by [78] as potential targets in cancer therapy. However, other anti-cancer strategies such as the attack to protein degradation function by blocking PSMB2 proteasome component has been also uncovered by the presented methodology. In this case, our result suggests that this therapy should be more efficient in tumors where PPP6C is mutated.

## Conclusions

We have proposed by the present study a tool for phylogenetic inference of candidates for future experimental validation as drug targets in anti-cancer therapy. Once more, we stress that we do not argue in favor of a methodology of SL-genes inference across distant species, as it has been already discussed in the literature to be a controversial step [73,75]. Rather our study has a pharmacological utility and constitutes an alternative for massive drug screenings. In addition, the arguments brought forward in favor of the proposed candidates above justify their consideration for future experimental validation.

Furthermore, we provide an additional file on the results discussed above in order to foster the bioinformatic and pharmacological communities towards further analysis of this methodology.

## Abbreviations

SL: Synthetic Lethal; iHSLN: inferred Human Synthetic Lethal Network; YSLN: Yeast Synthetic Lethal Network; GO: Gene Ontology; HGNC: HUGO gene nomenclature committee.

## Authors' contributions

NCP performed database manipulation, graph construction and drafted the manuscript. AM and CRC wrote the manuscript, conceived and designed the investigation. AM contributed to computational analysis and CRC performed the biological interpretation. RVS contributed to manuscript preparation and promoted the work. All authors have read and approved the final manuscript.

## Supplementary Material

Additional file 1 **Detailed information on the statistical analysis of iHSLN biological functions**. File in a .xls format. Click here for file

Additional file 2 **List of 1078 human genes homologous to the 2383 SL yeast genes from the BioGRID database **(Figure 5). File in a .xls format. Click here for file

Additional file 4 **List of SL relations between the inferred human SL genes (iHSLN)**. File in .sif format to be loaded in cytoscape software. Edges are represented in two columns separated by the label pp. Click here for file

Additional file 5 **The network of SL relations between the inferred human SL genes (iHSLN) **(Figure 6). File in .cys format. This file must be opened with cytoscape software. Click here for file

Additional file 6 **Cancer-related genes and their first neighbors from the SL human network **(Figure 7). File in .cys format. This file must be opened with cytoscape software. Click here for file

Additional file 7 **An extract of SL-inferred human orthologs organized by GO numbers and related with DNA damage and cell-cycle **(Figure 8). File in .cys format. This file must be opened with cytoscape software. Click here for file

Additional file 3 **Sub-network of SL nodes that are also drug targets, and their first SL neighbors**. File in .cys format. This file must be opened with cytoscape software. Click here for file

Additional file 8 **Drug-cancer SL network representing the set of cancer-related nodes and their SL drug-associated partners **(Figure 9). File in .cys format. This file must be opened with cytoscape software. Click here for file
